# Immune Response in the Liver under Conditions of Infection, Malignancy, and Transplantation

**DOI:** 10.1155/2014/709126

**Published:** 2014-02-06

**Authors:** Basak Kayhan, Sukru Oguz Ozdamar, Winfried Padberg, Uner Kayabas, Rina Aharoni, Saied Mirshahidi

**Affiliations:** ^1^İnönü University, Liver Transplantation Institute, Department of Immunology, 44100 Malatya, Turkey; ^2^Zonguldak Bülent Ecevit University, Faculty of Medicine, Department of Pathology, 67600 Zonguldak, Turkey; ^3^Department of General, Visceral, Thoracic, Transplantation and Pediatric Surgery, University Hospital Giessen, Rudolf-Buchheim Street 7, 35395 Giessen, Germany; ^4^İnönü University, Faculty of Medicine, Department of Infectious Diseases and Clinical Microbiology, 44100 Malatya, Turkey; ^5^The Weizmann Institute of Science, Immunology Department, 76100 Rehovot, Israel; ^6^Department of Medicine & Basic Science, LLU Cancer Center Biospecimen Laboratory, Loma Linda University, Loma Linda, CA 92350, USA

The liver is the largest solid organ in the body and commands a large blood supply that is rich in bacterial products, environment toxins, and food antigens, which are repeatedly scanned by cells within the liver. In addition, the regenerative capability of liver tissue makes it unique in comparison to other internal organs. In contrast to these unlike properties, the liver is one of the most common sites for metastatic diseases; it supports chronic viral infections caused by hepatitis B and C and can also promote tolerance against external antigens in animal models. Furthermore, liver transplantation often requires less immunosuppression compared to kidney and other solid organ transplantations. Despite these properties, the study of liver immunology remains in its infancy, and many questions remain unanswered. In that special issue, authors try to find answers in various subjects about immune response in liver under the approach of infection, malignancy, and transplantation.

In case of research articles, T. Nakano et al. aimed to clarify the role of autoantibodies in autoimmune hepatitis on liver allograft rejection and acceptance. Additionally they investigated whether there is a relationship between the elevation on anti-nuclear antibodies and the function of regulatory T cells in this phenomenon. In another research, F. Miao et al. try to find an association of HLA polymorphism in spontaneous clearance of hepatitis B Qidong Han population in China by genotyping HLA-A, -B, and -C loci of individuals with HBV persistent infection and individuals with spontaneous clearance of HBsAg.

It is well known that anti-HLA antibodies and antibodies against major histocompatibility complex (MHC) class I-related chain A (MICA) antigens are crucial in renal transplantation not only during pre-op but also in post-op periods. However, we have less amount of knowledge about the role of these antibodies on liver transplantation. M. Ciszek et al. from Medical University of Warsaw clears that question in their long-term survey. In an interesting observation performed by T. V. Sharkova et al. immunomorphologic manifestations in liver and liver failure during highly pathogenic H5N1 avian influenza virus infection in mice were presented. Y. Nobuoka et al. address the usefulness of monitoring immunological aspects by IMK assay CYP3A5 genotype assay not only in living donor liver transplantation but also in renal transplant patients.

Most cases of hepatocellular carcinoma (HCC) patients are detected in their late stages and become ineligible for surgical resection. It is known that more than 50% of HCC patients have persistent hepatitis B virus infection. In order to improve early diagnosis N. Pan et al. have used the knowledge of natural killer (NK) cell's important role on controlling viral hepatitis and its consequences on the pathogenesis of liver injury and inflammation. Since persistent inflammation has been recognized as a driving force in HCC genesis, N. Pan et al. assume that NK cell activation may also be involved in HBV-related HCC development. At that point we know that killer cell immunoglobulin-like receptors (KIR) are involved in the regulation of NK cell activation through recognition of their human leukocyte antigen (HLA) class I ligands. N. Pan et al. try to answer whether the KIR and HLA genetic background could also influence the onset age of HBV-related HCC or not.

Besides those research articles, authors discuss the immunological reactions and the role of different factors in liver diseases and liver transplantation in review articles. Concerning that, B. Liu et al. address the role of chemokines in chronic liver allograft dysfunction pathogenesis and potential use of these chemotactic factors as therapeutic agents. In another review, E. C. Cunningham et al. discussed the development of tolerance in liver transplant patients and marked dose effect theory and its use in clinics by combining a novel gene therapy. In case of tumor progression and metastasis, M. Pancione et al. suggest new hypotheses that favour the growing impact of tumor infiltrating cells on tumor progression, acquisition of different markers on tumor cells to enhance the interaction with tumor microenvironment, and their effect on therapy.

Overall we believe that this special issue will be beneficial for clinicians and all scientists working on liver immunology.

*Basak Kayhan* *Basak Kayhan* *Sukru Oguz Ozdamar* *Sukru Oguz Ozdamar* *Winfried Padberg* *Winfried Padberg* *Uner Kayabas* *Uner Kayabas* *Rina Aharoni* *Rina Aharoni* *Saied Mirshahidi* *Saied Mirshahidi*

